# Spatial distribution of nicotine concentrations in Berlin’s surface waters and their potential sources

**DOI:** 10.1007/s11356-025-36124-w

**Published:** 2025-02-27

**Authors:** Markus Venohr, Christine Beusch, Tobias Goldhammer, Hanh Hong Nguyen, Simone Podschun, Claudia Schmalsch, Christian Wolter

**Affiliations:** 1 Leibniz Institute of Freshwater Ecology and Inland Fisheries, Department of Ecohydrology and Biogeochemistry, Justus-von-Liebig-Str. 7, 12489 Berlin, Germany; 2 Faculty of Biology, University of Duisburg–Essen, Universitätsstrasse 2, 45141 Essen, Germany; 3 University Alliance Ruhr, Research Center One Health Ruhr, Essen, Germany; 4 Leibniz Institute of Freshwater Ecology and Inland Fisheries, Department of Fish Biology, Fisheries and Aquaculture, Müggelseedamm 310, 12587 Berlin, Germany

**Keywords:** Cigarette butts, Urban surface water, Emission sources, Wastewater treatment plants, Sewer effluents, Storm water events, Emergent pollutant, Effects on aquatic organisms

## Abstract

Nicotine is a ubiquitous emergent pollutant that primarily enters the environment through inappropriate disposal of cigarette butts. In a 7-week monitoring program, we collected 56 water samples from 14 lakes, 9 ponds, 9 rivers, 8 canals, and 2 canalized brooks in Berlin. Nicotine was detected in all investigated surface waters. Observed concentrations ranged between 7 ng/l and 1469 ng/l (mean 73 ng/l, median 28 ng/l). Rainy weather conditions generally led to an increase in nicotine concentrations, particularly in canals where concentrations were up to 16 times higher after rain events. For water bodies receiving sewer discharge, mean nicotine concentrations were positively related to population density, while concentrations in surface waters without sewer connections were more related to the presence of public transport stops or recreational areas. Our results highlight the high spatiotemporal variability of nicotine concentrations in urban surface waters. We recommend a temporary systematic daily or event-based monitoring of nicotine concentrations to support our findings and to better understand and quantify emission sources and concentration decay phases. This would improve our still incomplete knowledge about ecological impacts arising from long-term below-lethal nicotine concentrations in urban surface waters.

## Introduction

Nicotine is widely consumed via tobacco products, particularly cigarettes, and is a ubiquitous toxin in our environment (Asadi et al. 2023; Seckar et al. 2008). The effects of cigarette smoking and nicotine on human health are well studied, but little is known about nicotine concentrations in surface waters and the associated ecological risks (Buerge et al. 2008; Oropesa et al. 2017).

Globally, 5.7 trillion cigarettes were consumed in 2016 (Drope et al. 2018), of which 76% to more than 90% are inappropriately disposed in the environment (Ghasemi et al. 2022). Consequently, cigarette butts are reported to be the most common litter in urban areas (e.g., Novotny and Slaughter (2014) and Torkashvand et al. (2020)) and a dominant waste source at marine beaches (Ocean Conservancy 2020). Additionally, although trends in cigarette consumption vary internationally (Kloft et al. 2020), cigarette butt litter is likely to increase further given the number of smokers is projected to increase in the future (Ghasemi et al. 2022). Aside from consumption trends, the density of littered cigarette butts is commonly high in urban districts, in particular near hospitality venues, public transport hubs, and entrances to educational facilities and playgrounds (Valiente et al. 2020) or at recreational and tourist areas during holidays and warm seasons. Densities of littered cigarette butts can also be higher in areas that are not regularly cleaned, e.g., canals (Ghasemi et al. 2022). In addition to nicotine, cigarette butts contain a cocktail of substances, such as polycyclic aromatic hydrocarbons (PAHs) and heavy metals, e.g., arsenic (Montalvao et al. 2019; Roder Green et al. 2014), some of which can be highly toxic to aquatic organisms (Torkashvand et al. 2020). These substances can originate from a variety of sources (e.g., traffic or industry), but there are very few nicotine sources other than littered cigarette butts, as albeit nicotine can originate from tobacco plant production and processing (Torkashvand et al. 2020) and from atmospheric deposition. Nicotine therefore serves as a tracer for domestic wastewater. One cigarette contains an average of 17.1 to 18.7 mg/g of nicotine (equaling 11.9–14.5 mg per cigarette; Roder Green et al. 2014), which is largely inhaled during smoking. The partially exhaled nicotine leads to an almost ubiquitous occurrence of nicotine in the atmosphere, the concentration of which increases with population density and becomes deposited on land and water surfaces with precipitation as humid atmospheric deposition (Aquilina et al. 2021). However, the available data for this pathway are too limited to be considered further in this study.

Nicotine entering the human body by smoking is metabolized mainly to primary metabolites nicotine N′-oxide (4–7%) and cotinine (70–80%), which are then further metabolized, leaving only 10–15% of cotinine excreted unchanged (Verovšek et al. 2022). Butts of smoked cigarettes retain on average 2.1 mg of nicotine (Roder Green et al. 2014), which can become 3.8 mg/l of nicotine on average in solution (extracted from 5 g cigarette butts and 50 ml solution after 2 h; Moriwaki et al. 2009). According to Verovšek et al. (2022), nicotine concentrations at wastewater treatment plant influents of up to 424,000 ng/l were found, which were reduced to 15–32,000 ng/l in wastewater effluents (Verovšek et al. 2022) or up to 600 ng/l as reported by Buerge et al. (2008).

Despite the relatively good elimination of nicotine in wastewater treatment plants, they are still an important source of nicotine to the environment (Baker and Kasprzyk-Hordern 2013). In some cases, higher concentrations of nicotine have been observed in the absence of, or upstream of, a wastewater treatment plant (Archer et al. 2017; Styszko et al. 2021), suggesting other relevant sources, such as untreated wastewater, e.g., from separate sewer systems or combined sewer overflows. Driven by the density of cigarette butts littered in the adjacent catchment, concentrations of released eluates in surface waters further depend on river flow, dilution, degradation and sorption processes, and sampling time, showing a seasonal variation (Verovšek et al. 2022).

About half of the nicotine in cigarette butts is dissolved by rain water after just 30 min (Roder Green et al. 2014). While leaching of nicotine and other substances from cigarette butts decreases over time, the filter itself and its related environmental risks remain for a long time (Ghasemi et al. 2022). Under laboratory conditions, eluate nicotine concentrations decreased from unused and overused cigarette filters with tobacco to filters without tobacco (Slaughter et al. 2011).

In most natural waters, the monoprotonated form of nicotine dominates (Beutel et al. 2021). The free-base form increases under alkaline conditions, is well water soluble (Li et al. 1992; Seckar et al. 2008), and can be assimilated by organisms (Benowitz et al. 2009). There is limited information regarding the abiotic and biotic transformations of nicotine in the environment. Photolytical degradation of nicotine by UV_254_ light can cause an almost complete reduction of nicotine in surface waters within 60 min, which is less complete with decreasing pH values and in presence of humic substances (Alberti et al. 2021). Under laboratory conditions, half-life values of monoprotonated nicotine (pH 6.5–7.0) ranged from months to a year (Passananti et al. 2014). For aerobic soil slurries, Beutel et al. (2021) cited from a report by the R.J. Reynolds Tobacco Company that nicotine biodegradation has a half-life of ~ 3 days. Nicotine tends to adsorb to charged surfaces such as bentonite clays (Hakoyama et al. 2000), which could support the result by King et al. (2021) who found nicotine metabolites more than 60 days after exposure to marine sediments, indicating possible long-term effects on benthic fauna.

In the past, nicotine was commonly used as natural insecticide against herbivorous insects, but was later banned and replaced by synthetic neonicotinoids. This already suggests the potentially threatening ecological effect of nicotine. Toxic lethal effects have been reported on mudflat snails (Booth et al. 2015), freshwater invertebrates (Green et al. 2020), fathead minnows (Slaughter et al. 2011), other freshwater fish species (Konar 1980), and *Daphnia* sp. (Oropesa et al. 2017). At lower concentrations (≥ 10 μg/l), nicotine acts as a weak juvenoid compound in daphnia (*Daphnia magna*), increasing the proportion of male offspring, while higher concentrations (100 μg/l) impair daphnia reproduction by reducing offspring numbers and body sizes (Oropesa et al. 2017).

While studies commonly focus on the effects of nicotine on multicellular organisms, Yang et al. (2020) reported nicotine to be antimicrobial because many microbes, such as bacteria (Mu et al. 2020) and fungi (He et al. 2019), can metabolize nicotine (Beutel et al. 2021). Baran et al. (2020) mentioned the potential effects on unicellular microorganisms including bacteria and potential effects on aquatic ecosystems or sludge activity in wastewater treatment plants, respective of their treatment efficiency.

In recent years, the number of studies on effects from littered cigarette butts has steadily increased (Dobaradaran et al. 2021). These studies mostly focused on the mixture of various chemicals released from cigarette butts and the degradation or ecological effects of filters. Due to the relatively fast transformation of nicotine and the easier chemical analytics, studies on the dominating metabolites cotinine and trans-3′-hydroxycotinine (Benotti and Bronnawell 2007) are more common. As a consequence, occurrence, fate, and transport of nicotine in surface water are not well studied (Beutel et al. 2021).

As carelessly littered cigarettes are a main source of nicotine (Roder Green et al. 2014) released to the environment, it can be assumed that butts accumulate during phases without precipitation and that increased nicotine concentrations are carried into rivers and lakes especially during a first precipitation event (first flush). This effect should be particularly pronounced when sewer systems discharge nicotine-enriched stormwater into surface waters. Therefore, in this study, nicotine concentrations were measured in standing and flowing waters of different sizes in a densely populated city Berlin (Germany), with and without connected sewer systems, but partly with adjacent lawns and beach resorts. We addressed the following research questions:

1. How do nicotine concentrations differ in different types of surface waters?
2. Can changes in nicotine concentrations be explained by storm water events?
3. What are the controlling variables of nicotine concentrations (e.g., connected sewer systems, land use, or population density)?
4. Do the observed concentrations pose a potential risk of short- or medium-term damage to aquatic organisms?

## Materials and methods

### Study area

Berlin is in the north-eastern lowlands of Germany. It covers an area of 891 km^2^ and has 3.7 million inhabitants (Berlin-Brandenburg Statistics 2023), resulting in an average population density of 4119 inhabitants/km^2^. According to BKG (2018), the current sealed urban area is 216.5 km^2^, which corresponds to a population density of 16,952 inhabitants/km^2^ sealed area.

Berlin is in one of the driest areas of Germany, receiving on average only 570 mm/m^2^/year precipitation (Cornes et al. 2018). Three larger rivers flow through the city: River Havel and its lake-like extensions 27.1 km from North to South, River Spree 45.1 km from East to West entering the River Havel, and River Dahme 16.4 km joining river Spree in the south-east of the city. Further, a total of 80.1 km of navigable canals cross the city, including Teltowkanal in the South, Landwehrkanal in the center, and Berlin-Spandauer-Schifffahrtskanal and Westhafenkanal in the North.

**Fig. 1:**
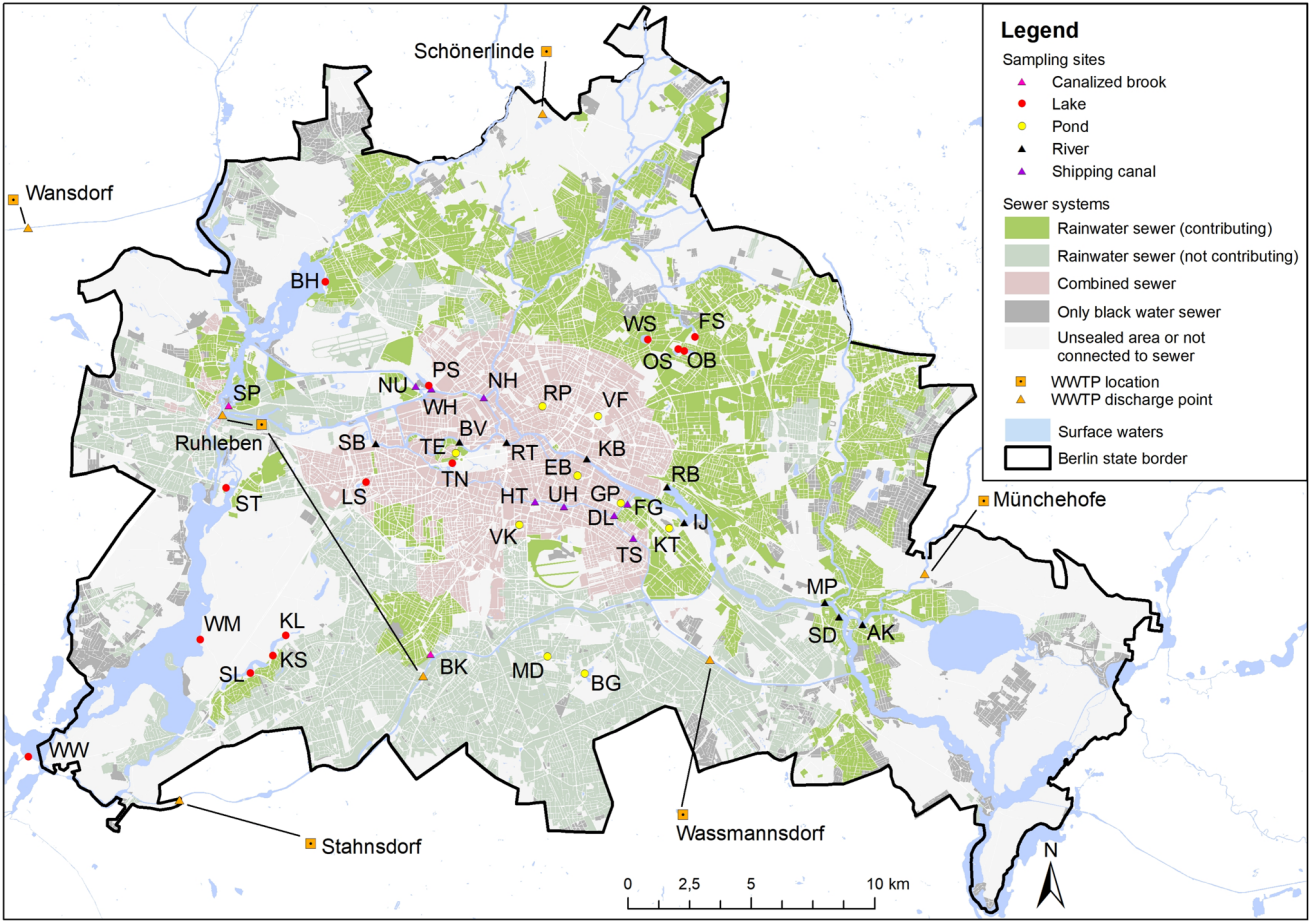
Location of sampling sites, types of sewers, and waste water treatment plants in Berlin. Information on the sewer discharge points (Fig. 6 in the Appendix), the sewer catchments (Fig. 7 in the Appendix), and the discharge contribution to receiving waters (Tables 6 and 7 in the Appendix) are provided in the Appendix

Six large wastewater treatment plants (Schönerlinde, Münchehofe, Waßmannsdorf, Stahnsdorf, Ruhleben, Wansdorf) treat Berlin’s wastewater, about 255·10^6^ m^3^ in 2019 (Bathe et al. 2021) (Fig. 1). The sealed urban areas are connected either to the combined sewer system, which is mainly located in the old city center, or to the separate sewer system (Fig. 1). In Berlin about 54·10^6^ m^3^ rainwater is directly discharged into surface waters (5.5·10^6^ m^3^ of them via the mixed sewer system) each year (Wicke et al. 2021), which washes cigarette butts, and consequently nicotine into the waters. The rainwater sewer system of the separate sewer system operates mostly by gravity and discharges water from streets and other sealed urban areas at various discharge points across the city. Combined sewers, which collect rainwater and wastewater from households together, are also equipped with pumps that transport water between pipe systems. During dry weather, all wastewater from combined sewers is treated in one of the wastewater treatment plants without much delay. During larger precipitation events, rainwater can exceed the treatment capacity of the connected wastewater treatment plants. The untreated wastewater is held-back in additional storage facilities and, if the storage volume is exceeded, excess wastewater is discharged directly into surface waters, such as the Spree, Havel, and Teltowkanal, in so-called combined sewer overflow events. These events are weather-dependent and vary considerably from year to year. For example, between 2007 and 2017, the number of annual overflow events ranged from 33 to 60, and the amount of water discharged ranged from 2.1 to 7.5·10^6^ m^3^ (Environmental Atlas 2024). Variability in the location and intensity of precipitation may also lead to locally different frequencies of overflow events and amounts of untreated released wastewater.

### Sampling sites

Sampling sites were selected to represent a wide range of water body types and local conditions of the city of Berlin. Five different water body types were monitored: rivers, canals, canalized brooks, lakes, and ponds (Tables 1 and 4 in the Appendix). Compared to lakes, ponds are in general smaller and not connected to other surface waters. First, water bodies with (rivers, canals, and some lakes) and without (ponds and some lakes) connections to sewer systems were selected (Table 1, Figs. 6 and 7 in the Appendix). Further, we selected water bodies according to the surrounding population density.

**Table 1:** Overview of water body types (WBT) and considered sampling sites with and without connections to sewer systems. The area data for ponds and entire lakes (ELs) refer to the entire water surface area, while for rivers and canals an area of 1 km upstream and for parts of lakes (PLs) a buffer of 1 km from the sampling point is considered. The full names of the sampling sites are given in Table 4 in the Appendix

WBT	No. of sampling sites (short names)	Area in ha			
		Min	Max	Mean	Total
River (with)	9 (RT, BV, SB, KB, AK, MP, RB, SD, IJ)	5.4	31.4	16.6	149.1
Lake (with)	EL: 4 (FS, OB, SL, ST) PL: 2 (KS, BH)	1.7	108.8	29.0	174.3
Lake (without)	EL: 5 (LS, OS, PS, TN, WS) PL: 3 (KL, WW, WM)	1.0	428.4	115.0	919.6
Pond (without)	9 (RP, VK, GP, TE, VF, MD, EB, KT, BG)	0.1	8.9	1.7	15.2
Canalized brook (with)	2 (BK, SP)	0.4	16.3	8.4	16.8
Canal (with)	8 (DL, FG, HT, NH, NU, TS, UH, WH)	2.9	11.6	5.4	42.9

Two sampling sites, Rummelsburger Bucht (RB) at River Spree and Landwehrkanal (TS), were sampled every week during the study period (15.05.2019 to 04.07.2019) as they are located in highly populated areas and connected to sewer systems; thus, we expected them to have elevated nicotine concentrations. RB is a bay partly separated from the River Spree, but with regular water exchange. The Landwehrkanal is characterized by very low flow velocities (Fig. 11 in the Appendix). It receives water only during sluice operation for navigation and from sealed areas in the catchment of connected sewers during rain events. All other sites were sampled once or twice to assess spatial patterns and variability of nicotine concentrations.

### Sampling and analysis

Surface water sampling was conducted between 15.05.2019 and 04.07.2019. Duplicate grab water samples of approx. 1000 mL were collected from the water surface in the morning, directly brought to the lab, and immediately processed to reduce nicotine losses by biological degradation. Pre-processing of the samples (e.g., filtering, weighing, adding labelled nicotine) occurred as soon as possible and analysis of nicotine through high-performance liquid chromatography and tandem mass spectrometry (HPLC-MSMS) was conducted in the morning of the subsequent day. Therefore, sampling was restricted from Monday to Thursday and to three sites, which could be processed per day.

The determination of nicotine concentrations followed a multi-step protocol according to Martínez Bueno et al. (2010) and Valcarcel et al. (2011). Reagents included ultra-pure water (Honeywell), acetonitrile (ACN, Roth), methanol (Roth), formic acid (Sigma–Aldrich, all LC–MS grade), ammonium acetate (Fluka, HPLC grade), ammonium hydroxide solution (Honeywell), and l-nicotine (99 + %, Acros Organics). First, water samples of approximately 350 ml were filtered with a pre-combusted glass fiber filter (Whatman GF/F) to remove solid particles. The pH was adjusted to 8 by adding ammonium hydroxide. Then, 250 ml of this filtrate was spiked with 3 ng deuterium-labeled nicotine (nicotine-D4, Sigma Aldrich) as an internal standard for quality assurance during sample preparation and measurement.

The sample was concentrated using a solid-phase extraction column (SPE, OasisTM HLB Extraction Cartridge, 200 mg, 6 cc, Water Inc.) because concentrations were typically too low for direct measurement. The cartridges were conditioned with 6 ml of methanol and 6 ml of ultrapure water with pH 8. After drying the extraction columns, the sample was subsequently eluted with 5 ml methanol. The total amount of eluate was reduced in vacuo almost to dryness and the sample was again redissolved with 250 µl of water.

This purified sample was then deployed in the instrumental analysis using high-performance liquid chromatography and tandem mass spectrometry (Agilent 1290 Infinity II, 6470 QQQMS). Chromatographic separation of nicotine and nicotine-D4 was performed on a HPLC column (Poroshell 120EC-C18, 2.1 × 100 mm, 2.7 μm, 55 °C, with a gradient of 0.1% formic acid and 5 mM ammonium acetate (A) and acetonitrile (B) at 0.4 ml min^−1^, 1 µL injection volume). Ionization was performed by electron spray in positive mode, and mass spectrometric measurement was performed as multiple reaction monitoring for a concentration range of 0.5 to 30 ng/ml. Samples above this measurement range were diluted and measured repeatedly. The analytes were quantified using external calibration standards for nicotine and nicotine-D4 in the range from 0.5 to 30 μg/l that were prepared in a matrix of 80:20 of ultrapure water and ACN. The limit of quantification was set at 0.25 μg/l and the limit of detection at 0.1 μg/l. The linearity of nicotine was *R*^2^ = 0.9996 and of nicotine-D4 *R*^2^ = 0.9992. The accuracy for nicotine was 3.2% and the precision was 0.4%, determined in repeat measurements of a 10 µg/l nicotine standard (*n* = 10). A calibration sample was measured every day (see Tables 8, 9 and 10 in the Appendix for calibration and operation parameters). Blanks (ultrapure water) and control standards (10 and 30 µg/l nicotine and nicotine-D4 in 80:20 water:ACN) were measured after six samples.

### Catchment data for source analysis

Information on the type of sewer system for the year 2017 was provided by FIS-Broker (2022). For separate sewer systems, the connected urban area (sewer catchment area) and the receiving surface water body, specified as section of rivers and canals, were provided. For combined sewers, FIS-Broker (2022) provided only general information on connected areas, with no indication of discharge points or receiving waters. Information on the location of a total of 109 combined sewer discharge points as well as the connected areas was provided by the Senate of Berlin (personal communication with Michael Wagner). However, for overflow events, the discharge is released predominantly via 32 outlets, for which corresponding flow shares were provided by the Berliner Wasserbetriebe treating Berlin’s wastewater (personal communication with Erika Pawlowsky-Reusing). Catchments and receiving surface waters were derived from areas connected to the different combined sewer systems. Unsealed or unconnected urban areas, also provided by FIS-Broker (2022), were excluded for all subsequent calculations. In addition to the sewer catchments, a 100-m buffer was considered along the sampled water bodies. For rivers, canals, and lake-like river sections (Table 1), the buffer was formed 1 km upstream from the sampling site or, for lakes and ponds, around the sampled water body.

Green et al. (2020) reported very high densities of discarded cigarette butts at bus stops and entrance areas to subway stations. As an indicator for potentially increased nicotine concentration, we extracted locations of public transport stops (metro, subway, bus, tram) from Open Street Map (OSM 2021), assuming that public transport type does not correlate with littered cigarette butt densities.

The land use information was taken from the land-cover map scale 1:25,000 (BKG 2018) transformed into a 10-m grid using the land use attribute “LN_AKT.” This attribute distinguishes 88 different land use types that have been aggregated into six groups, representing relevant urban land uses: residential, urban green spaces, sports and leisure, roads and public transport, public areas, production, and other.

Population density, precipitation, runoff, public transport stops, and land use were considered factors that may affect nicotine concentrations. Population density was provided by FIS-Broker at the house block level for 2017. Daily precipitation totals were extracted from a daily gridded map (0.1°/ ~ 11 km spatial resolution, E-OBS data) (Cornes et al. 2018). Data extraction from the EOBS file, which is in NetCDF format, was performed in R using the package “RNetCDF,” version 2.3–1. Information on the location of public transport stops was derived from OSM (2021). Observed discharges from three gauging stations on the Spree and Landwehrkanal were obtained from Water Portal Berlin (2024).

### Linking sampling sites and the connected sewer catchment

For a 100-m buffer around sampling sites and connected sewer catchments (if applicable), mean daily precipitation (for the sampling day and the previous 2 days), mean population density, and land use information were obtained using ESRI ArcMAP (versions 10.4) “zonal statistics.” For public transport stops, the “Spatial Join” function was applied with an additional search distance of 15 m, since many public transport stops were mapped on green/unsealed areas along roads.

The half-life of nicotine in water is about 3 days (Beutel et al. 2021; Seckar et al. 2008). The total flow distance from the outlet of Lake Müggelsee to the confluence with River Havel is about 45 km. Water in River Spree needs 7–14 days to pass Berlin, depending on the flow path and due to the longer residence times in canals, bays, lakes, and harbor basins. This corresponds to an average flow rate of about 3 to 6 km per day. This means that most of the nicotine that enters the River Spree at the entrance to Berlin is degraded before the Spree water leaves Berlin again. For our analysis, we considered discharge points of sewer systems with a maximum distance of 5 km (corresponding to 1 to 2 days) to obtain an observable influence from these emission sources. Similarly, we extracted daily precipitation sums for the day of sampling and the two preceding days, each for the coordinates of the sampling sites and as an area-weighted mean for the catchment of the connected sewers. In the case where two or more sewer catchments contributed to a sampling point, the sum of the population and public transport stops and the area-weighted average of precipitation were calculated.

Statistical analysis and figures were done with the software R (version 4.3.0), using the Spearman/Pearson correlation test and the packages ggplot2 (version 3.5.1), data.table (version 1.14.8), and dplyr (version 1.1.4). Maps were generated using the software ESRI ArcMAP (version 10.4).

## Results

### Mean nicotine concentrations and effects of catchment characteristics

**Fig. 2:**
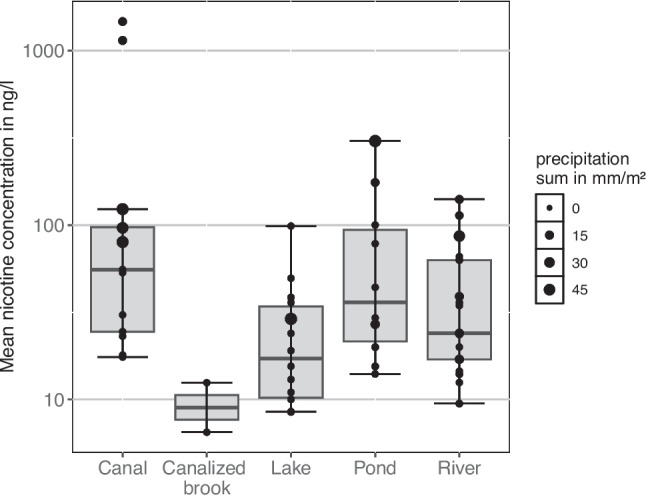
Range of observed nicotine concentrations in ng/l per water body type. The point size indicates the amount of precipitation in a 100-m buffer around a surface water body or (if applicable) in a sewer catchment

Nicotine was detected in all investigated surface waters. The mean nicotine concentration across all sampling sites and days was 73 ng/l with a median of 28 ng/l. The lowest nicotine concentration was 7 ng/l (sampling site: SD, 5/28/2019) and the two by far highest total concentrations were 1469 ng/l and 1144 ng/l (sampling site: TS, 5/21/2019 and 6/21/2019). Concentrations differed among water body types and were highest in canals (mean = 249 ng/l) followed by ponds (mean = 81 ng/l), rivers (mean 43 ng/l), lakes (mean = 26 ng/l), and canalized brooks (mean = 10 ng/l) (Table 2, Figs. 1, 2). Mean nicotine concentrations in water bodies receiving sewer discharge (110 ng/l) were twice as high as those in water bodies without connected sewer catchments (55 ng/l).

**Table 2:** Observed mean nicotine concentrations differentiated by weather conditions and water body type

Type of water body (with) and (without) connected sewer system	All samples		3-day precipitation sum = 0		3-day precipitation sum > 0		Conc change
	No. of samples	Conc in ng/l	No. of samples	Conc in ng/l	No. of samples	Conc in ng/l	Without units
River (with)	17	43.4	8	33.8	9	51.9	1.5
Lake (with)	6	32.3	6	32.3	0	-	-
Lake (without)	8	22.0	5	19.7	3	25.8	1.3
Pond (without)	10	80.7	3	74.0	7	83.6	1.1
Canalized brook (with)	2	9.5	2	9.5	0	-	-
Canal (with)	13	248.7	7	31.8	6	501.7	15.8
Total	56	72.8	31	29.4	25	147.9	5.0

### Effects of precipitation on nicotine concentrations

Hot dry summer days dominated the sampling period; 40 of the 56 samples were taken under these weather conditions. When including weather conditions of the 2 days prior sampling (3-day precipitation sum > 0 mm/m^2^), in total, 25 samples represent rain-influenced (rainy) conditions. The mean nicotine concentration of all sites was substantially lower at dry (33 ng/l) compared to rainy weather conditions (166 ng/l). This increase was found for all water body types (Table 2 and Fig. 4), being the strongest in canals, where nicotine concentrations increased about 16 times after rain. This increase was much lower for rivers with sewer connections (1.5 times) and lowest for ponds without sewer connection (1.1 times). Nicotine concentrations are not available for all combinations of water body type, with/without sewer connections, and at dry/rainy weather conditions because rain events were too rare to occur across all possible combinations.

Nicotine concentrations at sampling sites connected to sewer catchments showed a weak but significant correlation with the 3-day precipitation sum (*n* = 25, *r*^2^ = 0.17, *p* = 0.02, Fig. 8 in the Appendix). This can also be seen in Table 2, and when comparing maximum precipitation in the sewer catchment (Fig. 4, top-left, outer circle) with the observed concentration at rainy days (Fig. 4, bottom-right).

### Effects of land use on nicotine concentrations

According to the land use map (LBM-2018 provided by BKG (2018), 53% of the land in Berlin is either residential, roads and public transport, public areas, or production (Table 3). In the catchments of the canal sections, these land use classes cover 90% of the area. In contrast, in a 100-m buffer along rivers and lakes, urban green areas and sports and leisure areas occupy 40% of the area. The catchments of the sampled waters considerably differ in area shares with sport, recreation, urban green, and other uses, each covered up to 99% (Table 5 in the Appendix). The share of residential areas varies between 0 and 84%, while public places and production sites never exceed 24% and areas for streets and public transport always remain below 9%. No significant linear correlations were found between the observed nicotine concentrations and any land-use class or shares, even if differentiated for water body types or dry and rainy days.

**Table 3:** Percentages of the different land use classes in the 100-m buffer along the investigated surface waters, in the considered sewer catchment and in Berlin according to land use map LBM-2018. (BKG 2018). Table 5 in the Appendix further provides information on land use share per sampling site

Area shares [%] in the	Residential	Urban green spaces	Sports and leisure	Roads and public transport	Public areas	Production	Other
a) 100-m buffer	23.9	17.8	22.6	2.2	8.6	9.4	15.3
b) Sewer catchment	61.0	2.6	5.4	4.1	16.1	8.5	2.2
a) + b)	59.9	3.0	5.9	4.1	15.9	8.6	2.6
Berlin mean	36.5	5.5	10.0	2.7	7.9	5.5	31.9

### Effects of population density on nicotine concentrations

Across sampling sites and dates, we found no correlation between nicotine concentrations and population density in the 100-m buffer and the sewer catchments. When restricting the data set to only sampling sites receiving sewer discharges and rainy conditions, we found a weak but significant correlation between population density and nicotine concentrations (*n* = 13, *r*^2^ = 0.13, *p* = 0.1, Fig. 9 in the Appendix). Using all data, for population density classes of < 1000/km^2^, 1000–10,000/km^2^, and > 10,000/km^2^ mean nicotine concentrations increased from 21 to 54 to 65 ng/l, respectively. For sampling sites without connected sewer catchments, nicotine concentrations did not show any change with increasing population densities.

### Effects of public transport stops on nicotine concentrations

According to OSM (2021) Berlin has 14,273 public transport stops averaging 16 stops per km^2^. For all water bodies sampled, the public transport stop density in the 100-m buffer and, if applicable, in the sewer catchment only showed very weak correlation with observed nicotine concentrations (*n* = 56, *r*^2^ = 0.09, *p* = 0.01). However, sampling sites with a public transport stop density below Berlin’s average showed lower mean nicotine concentrations (40 ng/l) compared to those with an above average public transport stop density (63 ng/l). This correlation improved considerably when restricting the data set to rainy conditions (*n* = 20, *r*^2^ = 0.31, *p* = 0.01) (Fig. 10 in the Appendix).

### Change in nicotine concentrations with runoff

**Fig. 3:**
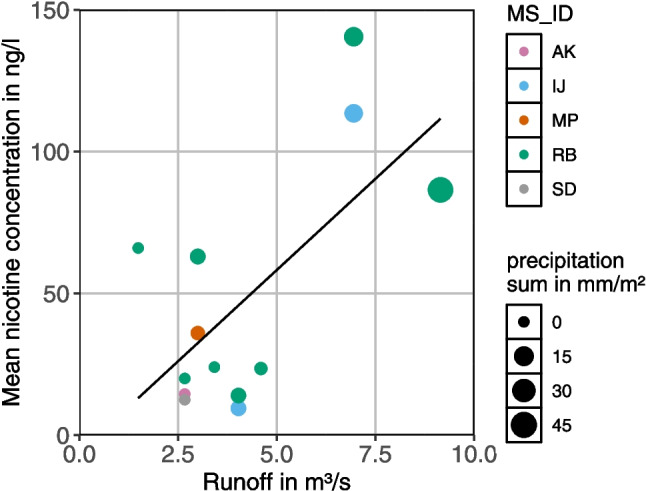
Correlation between the Allendestraße gauge discharge and the mean nicotine concentration at neighboring sampling points in River Spree (AK, Alt Köpenick; IJ, Insel der Jugend; MP, Mellowpark; RB, Rummelsburger Bucht; SD, Spree at confluence with Dahme). Point size indicates the sum of 3-day precipitation in the 100-m buffer and in the sewer catchment

In the Spree River, nicotine concentrations were significantly positively correlated (*n* = 13, *r*^2^ = 0.43, *p* = 0.001) with discharge at the Allendestraße gauge (Figs. 3, 11 in the Appendix). When examining data from individual sampling days, nicotine concentrations at Rummelsburger Bucht (RB) and Isle of Youth (IJ) increased with discharge and precipitation. At other stations where nicotine was measured only once, runoff, precipitation, and nicotine concentrations were at low levels. For the nicotine sampling sites in River Spree and Landwehrkanal (here discharge variation is kept to a minimum by sluices, Fig. 11 in the Appendix), no significant correlations were found with discharge at the neighboring gauges Sophienwerder and Landwehrkanal Zoo, respectively.

### Spatial pattern in nicotine concentrations and effects of site characteristics

**Fig. 4:**
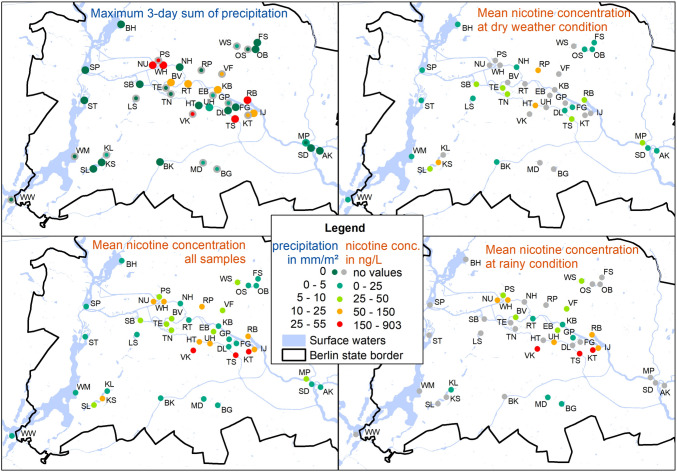
Top-left: Maximum sum of 3-day precipitation at the sampling site (inner circle) and in the sewer catchment (outer circle) and bottom-left: nicotine concentrations as mean of all samples and top-right, under dry, and bottom-right, rainy conditions per sampling site

In general, nicotine concentrations increased in the central city areas and remained low in the suburbs (Fig. 4, bottom-left). This pattern might have been driven by precipitation (Fig. 4, top-left). Except at Weissensee (WS), rain events were only captured for sampling sites in the city center. For dry days, mean nicotine concentrations were considerably lower than in rainy conditions (Table 2), though a slight increase towards the city center was still evident (Fig. 4, top-right compared to bottom-right). This spatial trend corresponds to higher population densities towards the city center. Sewer catchment area and the absolute number of inhabitants in the catchment (population density times catchment area) showed no significant correlation with nicotine concentrations in receiving water bodies. Public transport stop densities, however, were associated with slightly higher nicotine concentrations, and exhibited a strong positive correlation with population density in the considered sewer catchments (*n* = 25, *r*^2^ = 0.62, *p* < 0.001). Krumme Lanke south (KS) was the only sample in the suburbs with increased concentrations in dry weather conditions. This site is intensively used for bathing, recreation, and partying, which could be associated with a higher density of littered cigarette butts.

**Fig. 5:**
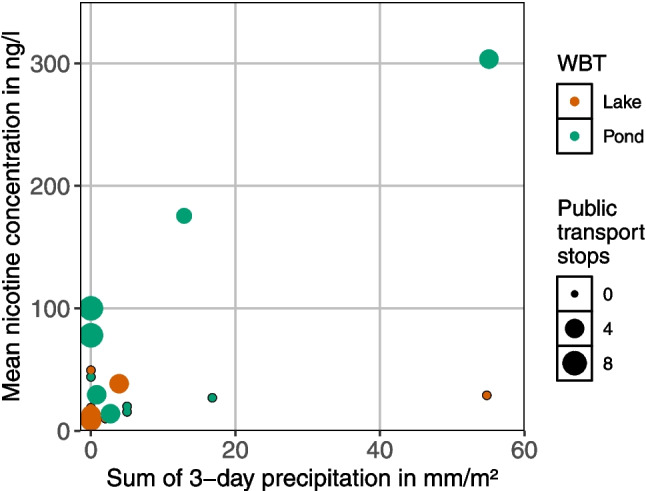
Correlation between precipitation and mean nicotine concentration for surface waters not connected to a sewer catchment. Point size indicates density of public transport stops in a buffer of 100 m and in the sewer catchment

**Fig. 6:**
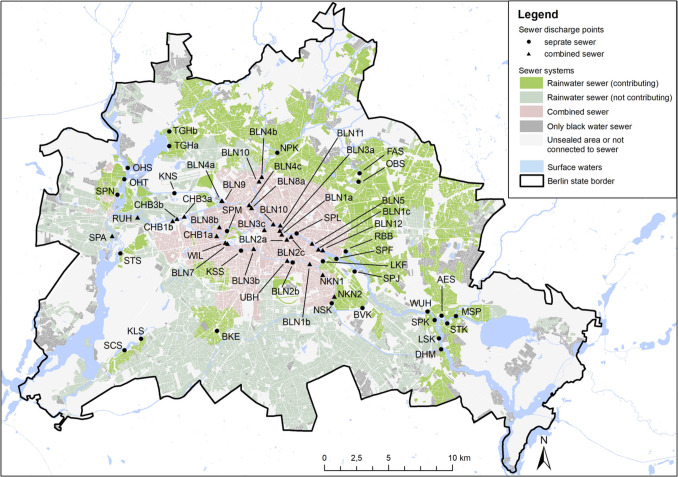
Sewer discharge points

**Fig. 7:**
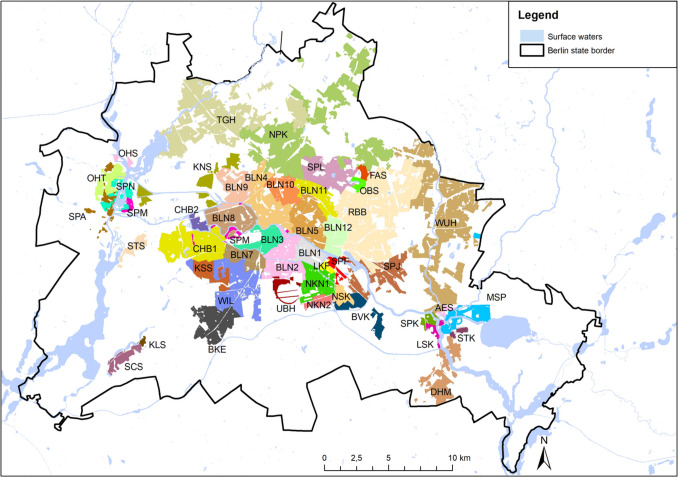
Catchment of considered sewer systems

**Fig. 8:**
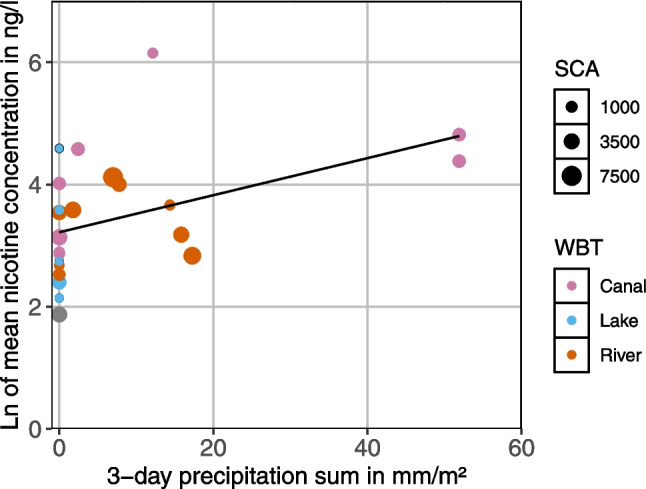
Ln of mean nicotine concentration at sampling sites connected to a sewer catchment in dependency from 3-day precipitation sum > 0 mm/m^2^ (*n* = 25, *r*.^2^ = 0.17, *p* = 0.02)

**Fig. 9:**
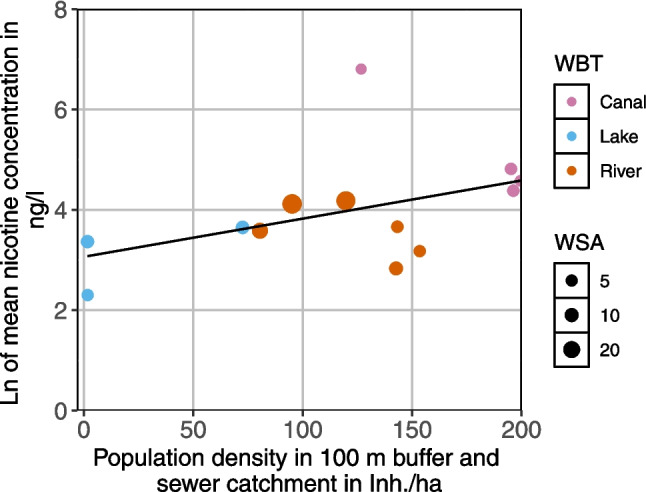
Ln of mean nicotine concentration per sampling site with 3-day precipitation sum > 0 mm/m^2^, excluding ponds, in dependency from population density (*n* = 13, *r*^2^ = 0.13, *p* = 0.1), distinguished for water body types (WBTs). Point size represents the surface water area in km.^2^ (WSA)

**Fig. 10:**
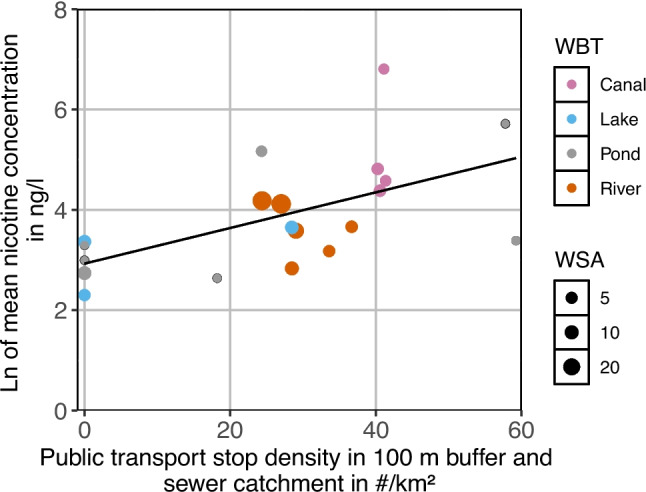
Ln of mean nicotine concentration per sampling site with 3-day precipitation sum > 0 mm/m^2^ and connected to a sewer catchment in dependency from public transport stop density (*n* = 20, *r*^2^ = 0.31, *p* = 0.01), distinguished for water body types (WBTs). Point size represents the surface water area in km.^2^ (WSA)

At sampling sites not receiving discharge from sewer catchments, nicotine concentrations were affected by the number of public transport stops combined with rainfall intensity. In dry weather, nicotine concentrations were low in lakes and ponds without connected sewer catchments, except when the number of public transport stops increased to a maximum. In rainy weather, concentrations increased with more public transport stops, but remained low when there were no stops (Fig. 5). However, this relationship is based on too few sampling sites to be statistically validated or generalized.

## Discussion

Nicotine was found in all water bodies studied ranging from 7 to 1469 ng/l, with a mean of 73 ng/l. However, even the highest concentrations measured here were in the lower range of the values reported by Wicke et al. (2021). Wicke et al. (2021) measured nicotine concentrations in different sections of the Berlin sewer system and below the outlet of wastewater treatment plants with median values (per site) between 360 ng/l and 2600 ng/l during precipitation events and with highest concentrations from individual measurements of up to 7900 ng/l.

Archer et al. (2017) reported nicotine concentrations of 154.3 ng/l and 245.5 ng/l, upstream and downstream of a wastewater treatment plant in the Gauteng Province of South Africa, representing an increase in nicotine concentration by a factor of 1.6. These reported concentrations indicate the importance of wastewater treatment plants and sewer systems as nicotine sources, even if our study only showed evidence for the latter.

Verovšek et al. (2022) conducted a comprehensive literature review on nicotine concentrations in different environmental compartments and reported maximum nicotine concentrations of 9340 ng/l for rivers. This maximum value likely derives from agricultural areas in Nigeria, where nicotine is used as a pesticide. The second highest value of 8187 ng/l was reported for a river in southern Poland upstream of a wastewater treatment plant, largely exceeding the values observed in this study or even those reported by Wicke et al. (2021). According to Verovšek et al. (2022), no observed nicotine concentrations were found for lakes, whereas mean concentrations of 432.6 ng/l were reported in 11 wetlands in eastern Spain.

The nicotine values found here are comparable to, but in the lower range of concentrations determined in Madrid (284 to 1885 ng/l, median 527.5 ng/l), probably because the sampling sites in Madrid were located closely downstream of sewage treatment plants (Valcarcel et al. 2011). It has not been clarified whether other factors, such as lower water flow in Spanish waters or higher proportion of smokers in the population, also had an influence. Even if there are some methodological differences in the laboratory measurement procedure, these cannot explain why values reported by Valcarcel et al. (2011) in drinking water are in some cases higher than those in surface waters in Berlin (with the exception of the Teltowkanal).

A broad survey of potable tap water samples from cities in Europe, Japan, and Latin America reported mean (maximum) nicotine and cotinine concentrations of 18 ng/l (305 ng/l) and 2 ng/l (14 ng/l), respectively (Boleda et al. 2011). Many of these tap water concentrations reported even exceed the surface water concentrations observed in this study.

However, concentrations observed in this study varied strongly depending on water body types, presence of connected sewers, and precipitation conditions. Population density and public transport stop density had only a weak effect on nicotine concentrations, but their influence was more pronounced under rainy conditions. Specifically, the correlation between population density and nicotine concentration is confounded by the overlap of high population density and the highest 3-day precipitation sums in the city center (Fig. 4, top-left). This spatial correlation makes it difficult to isolate the individual contributions of these factors to nicotine concentration increases, particularly given the relatively small dataset.

Although the influence of population density and public transport density is weak, the increase in nicotine concentrations is supported by findings of Green et al. (2020), who stated that public transport stops represent hotspots for discarded cigarette butts. However, this effect seems to be stronger when there are no sewer system catchments connected to the sampled water bodies and in combination with precipitation events.

The few precipitation events during the study period resulted in elevated nicotine concentrations. These elevated concentrations were detected not only on the day of precipitation, but also on the following days. The change in nicotine concentrations was stronger for waterbodies with connected sewer catchments and at low discharge conditions, i.e., in the canal (Table 2).

The effect of different rainfall events is not consistent. At Landwehrkanal (TS), precipitation of 12 mm/m^2^/day on 20.05.2019 was followed by the highest nicotine concentrations (1469 and 1144 ng/l) monitored here on two subsequent days, possibly caused by overflow events of connected combined sewer systems. However, on 12.06.2019, the 2-day precipitation total (thunderstorms on the previous two nights) was 43 mm/m^2^/day and nicotine concentrations remained at a medium level of 97 ng/l. This can be explained by varying (decreasing) nicotine concentrations in sewer effluents resulting from first and subsequent precipitation events or increased dilution of sewer effluents during high rainfall. Further, the reported strong photolytical degradation of nicotine by UV_254_ light (Alberti et al. 2021) might considerably reduce nicotine concentrations in surface waters if nicotine dissolved from cigarette butts is first exposed to UV light in puddles, before being flushed to sewers and surface waters.

**Fig. 11:**
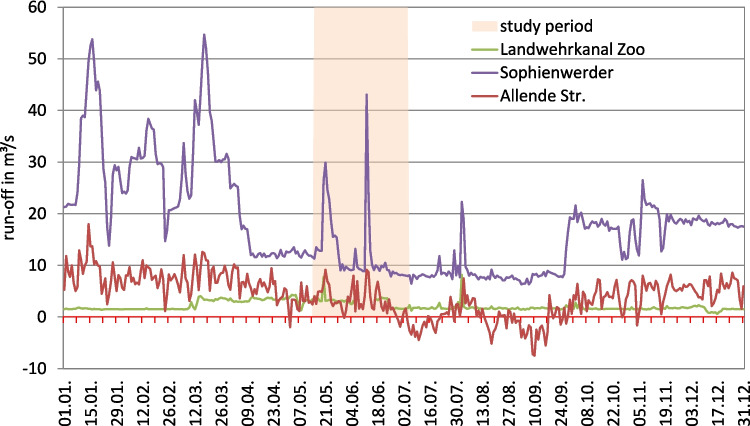
Observed runoff in 2019 and the study period at three gauging stations in Berlin

In Berlin, treated sewer effluents are only discharged into rivers, canals, or larger lakes, while smaller lakes and ponds do not receive sewer discharge. This is mainly done to ensure sufficient dilution of discharged sewage. In the Landwehrkanal, discharge is close to zero (Fig. 11 in the Appendix), which means that the emitted nicotine accumulates close to the sampling site, resulting in higher concentrations for the same inputs. In contrast, ponds and lakes without a connected sewer system can, in dry weather conditions, only be polluted by cigarette butts disposed of in the immediate vicinity, directly into the surface water, or by inflowing groundwater. However, this study did not consider groundwater as a discharge pathway. For ponds, locally discarded cigarette butts can cause elevated concentrations, which were visible at Carp Pond (KT) and Victoria Park (VK), but much less pronounced than in the Landwehrkanal. Thus, in dry weather, mean nicotine concentrations in lakes, rivers, and canals ranged from 27 to 30 ng/l, while concentrations in ponds, especially those without connected sewer systems, were more than twice as high.

During the study period, precipitation events considerably differed locally leading to (a) different amounts of precipitation between sampling sites and connected sewer system catchment on the same day, and (b) an unbalanced distribution of recorded precipitation amounts and events between water body types with and without connected sewer catchments. This inquires further investigations to enable direct comparison of observed nicotine concentrations between water body types and locations.

Although the presence of connected sewer systems impacted the level and variation of monitored nicotine concentrations, land use composition in the sewer catchments did not. This could be due to the fact that, with the exception of a few selected locations such as pubs, stadiums, or popular meeting places, cigarettes may be discarded in similar quantities and are not explicitly associated with a particular land use type, which might be too homogeneously distributed in the study area.

For Daphnia, Oropesa et al. (2017) and Valcarcel et al. (2011) derived predicted no effect concentrations (PNEC) of 100 ng/l and 2.4 ng/l, respectively. Concentrations affecting, e.g., reproduction of Daphnia, usually exceed 10,000 ng/l (weak juvenoid compound) or 100,000 ng/l (fewer and smaller offspring) as collated by Valcarcel et al. (2011). However, Konar (1980) reported 2000 ng/l for 168 h as a lethal concentration for Daphnia. Exposure time is of great importance in assessing the toxicity of nicotine. Vlasceanu et al. (2024) reported that the LC50 concentration of nicotine decreased by 93% when Daphnia were exposed for 48 and 24 h.

Further, Konar (1980) determined LC50 concentrations between 2210 and 8450 ng/l after contact for 168 h for ten freshwater fish species, which can be lowered to 500–5000 ng/l in the presence of sodium carbonate or lime. Lowest concentrations with a toxic effect have been reported as 120 ng/l for cladocerans and 2900 ng/l for fish by Valcarcel et al. (2011). From these values, it can be concluded that observed nicotine concentrations in Berlin’s surface waters, depending on the cited literature, partly exceed PNEC concentrations but hardly reach lethal values, neither in surface waters (this study) nor in sewer systems as reported by Wicke et al. (2021). An assessment of the toxicological relevance of the observed concentrations would require better knowledge of the long-term mean concentrations in surface waters. However, it can be assumed that relevant concentrations may only be found in or close to sewer or wastewater treatment plant outlets.

Missing assessment standards and still incomplete knowledge on environmental effects of nicotine on aquatic ecosystems correspond to a lacking official regulation for nicotine concentrations in surface waters. The substantially higher nicotine concentrations reported by Wicke et al. (2021) and Valcarcel et al. (2011) in the vicinity of wastewater treatment plants suggest that a temporary monitoring of nicotine in receiving surface waters could provide a better assessment of nicotine inputs and potential ecological effects.

In the absence of major production sites, nicotine enters the environment mainly through carelessly discarded cigarette butts. Since discarded cigarettes are not only an environmental and aesthetic litter problem but also a potential health hazard to humans (e.g., if they are thrown into the sandbox of a playground), eliminating the source seems to be the better approach than regulating wastewater treatment plants or sewer effluents. Many municipalities have therefore introduced fines, e.g., in Berlin, Germany, between €80 and €120 for discarded cigarette butts (Landesverwaltung Berlin 2019).

## Conclusion

Nicotine was detected in all investigated surface waters of Berlin. Concentrations vary depending on the type of water body, precipitation conditions, and the presence of connected sewer system catchments. Peak concentrations occurred particularly after rainfall events, with maximum concentrations generally higher at sampling sites receiving sewer discharge. Other characteristics of sewer catchments, such as population density, absolute number of inhabitants, or size of the sewer catchment, did not have a clear influence on nicotine concentrations. For sampling sites not receiving sewer discharges, local conditions were more important. However, it should be noted that the correlations found, their spatial pattern, and temporal differences are based on a relatively small number of observations and were generally weak, suggesting that follow-up studies should be carried out.

Future research should implement a higher (daily or event-based) sampling frequency and density, to detect the effects of individual precipitation events on the wash-off of nicotine eluates, including corresponding first-flush effects. Furthermore, the apportionment of wet atmospheric deposition should be assessed for a more complete quantification of sources. Additional research is necessary to ascertain decay rates and concentration decay phases. That is to say, the persistence of elevated concentrations in water bodies at differing water temperatures, and thus the identification of seasonal differences. Due to low dilution, frequently visited small standing inner-city surface waters should be particularly monitored because high concentration peaks can be expected. For the aforementioned reasons, the introduction of a temporary, systematic monitoring program for nicotine in surface waters is recommended.

Nicotine is a potentially toxic substance and knowledge about its transformation, accumulation in the food web, and effects on aquatic organisms is still limited. The concentrations measured in this study were predominantly well below the levels that could be toxic, or even lethal, to fish. However, they were partly exceeded for Daphnia (Oropesa et al. 2017; Valcarcel et al. 2011). There are significant gaps in the definition of monitoring methods and the systematic survey of LC50, as well as the effects of chronic sublethal nicotine concentrations on various developmental stages of groups of organisms. Beyond this, the comparability of the available studies is limited by the use of different experimental standards, the focus on a single cigarette brand (with or without filter), and the application of varying extraction and exposure times, as also noted by Green et al. (2022).

The environmental impact of discarded cigarette butts extends beyond nicotine and should be assessed together with other released toxic substances and microplastics. Despite the levying of fines, the discarding of cigarette butts is rarely prosecuted and continues unabated. Moroz et al. (2021) identified an efficient logistics system as the primary bottleneck in the collection of cigarette butts. Consequently, given the apparent political under-recognition of the direct costs associated with the cleaning of cigarette butt litter and its resulting ecological impacts, the introduction of a deposit system for cigarette butts could be both ecologically and economically viable due to their potential use as a recyclable material.

## Data Availability

All relevant data has been uploaded with the electronic supplementary material.

## Appendix

Figs. 6, 7, 8, 9, 10, and 11.

Tables 4, 5, 6, 7, 8, 9, and 10.

**Table 4:** Sampling sites, water body names, water body types (WBTs), and categorical information if sewer effluents are discharged max. 5 km upstream of the sampling site. *C* canal, *CB* canalized brook, *L* lake, *P* pond, *R* river

Short name	Water body name	Location	WBT	Sewer
AK	Spree	Alt Köpenick	R	Yes
BG	Hauptsee	Britzer Garten	P	No
BH	Tegeler See	Borsighafen	L	Yes
BK	Bäke	Bäke	CB	Yes
BV	Spree	Bellevue	R	Yes
DL	Landwehrkanal	Dreiländereck	C	Yes
EB	Engelbecken	Engelbecken	P	No
FG	Landwehrkanal	Flutgraben	C	Yes
FS	Fauler See	Lake outlet	L	Yes
GP	Görlitzer Parkteich	Görlitzer Park	P	No
HT	Landwehrkanal	Hallesches Tor	C	Yes
IJ	Spree	Insel der Jugend	R	Yes
KB	Spree	Katerblau	R	Yes
KL	Krumme Lanke	Lake inlet (north)	L	No
KS	Krumme Lanke	Lake outlet (south)	L	Yes
KT	Karpfenteich	Treptower Park	P	No
LS	Lietzensee	Bathing bay northern lake	L	No
MD	Eckernpfuhl	Alt-Mariendorf	P	No
MP	Spree	Mellowpark	R	Yes
NH	Westhafenkanal	Nordhafen	C	Yes
NU	Berlin-Spandauer-Schifffahrtskanal	Northern shore	C	Yes
OB	Obersee	West bay	L	Yes
OS	Orankesee	Bathing bay	L	No
PS	Plötzensee	Boat rental, south bay	L	No
RB	Spree	Rummelsburger Bucht	R	Yes
RP	Seerosenteich	Volkspark am Weinberg	P	No
RT	Spree	Reichstag	R	Yes
SB	Spree	Schlossbrücke	R	Yes
SD	Spree	Spreestr., height of river Dahme	R	Yes
SL	Schlachtensee	Height of train station	L	Yes
SP	Westlicher Abzugsgraben	Spandau	CB	Yes
ST	Stössensee	TU boathouse	L	Yes
TE	Teich am Teehaus	Tiergarten-Englisher Garten	P	No
TN	Neuer See	Tiergarten	L	No
TS	Landwehrkanal	Treptower Str	C	Yes
UH	Landwehrkanal	Urbanhafen	C	Yes
VF	Großer Teich	Volkspark Friedrichshain	P	No
VK	Wasserfall	Viktoriapark	P	No
WH	Westhafenkanal	Westhafen	C	yes
WM	Wannsee	Middle part	L	no
WS	Weissensee	South bay	L	no
WW	Wannsee	Western shore	L	no

**Table 5:** Percentage shares of different land-use classes in the 100-m buffer along studied surface waters and the connected sewer system catchment for the monitored surface waters according to land-use map LBM-2018 (BKG 2018)

Sampling site	Residential	Urban green spaces	Sports and leisure	Roads and public transport	Public areas	Production	Other
AK	64.3	1.9	3.5	2.6	20.4	4.5	2.9
BG	0.1	0.0	99.4	0.0	0.0	0.0	0.5
BH	72.7	2.2	3.5	1.1	10.7	8.5	1.3
BK	75.5	0.9	4.9	3.3	11.8	1.4	2.1
BV	53.5	4.5	5.0	6.0	20.5	9.4	1.0
DL	60.1	3.6	6.1	2.8	7.3	17.5	2.6
EB	59.0	20.6	0.0	0.0	15.4	0.0	5.0
FG	59.2	2.8	6.2	2.8	8.4	18.0	2.6
FS	67.9	10.4	1.4	1.7	13.9	0.1	4.6
GP	22.3	70.4	0.0	0.0	7.1	0.0	0.2
Hat	60.0	4.1	8.6	2.7	17.9	5.6	1.2
IJ	60.1	2.0	4.4	5.3	15.1	10.7	2.5
KB	49.0	2.3	5.7	7.4	19.5	13.9	2.2
KL	0.0	0.0	0.0	0.0	0.0	5.6	94.4
KS	60.0	0.0	0.0	0.0	4.7	0.0	35.4
KT	4.0	91.7	3.8	0.0	0.0	0.0	0.5
LS	39.8	0.1	52.1	1.0	6.7	0.0	0.2
MD	17.0	5.1	75.7	1.9	0.0	0.0	0.2
MP	73.1	1.9	3.4	3.2	11.1	4.4	2.8
NH	63.6	3.4	5.2	3.2	14.8	6.7	3.0
NU	60.9	2.6	7.2	3.9	17.9	5.0	2.5
OB	84.0	2.5	8.2	1.9	3.3	0.0	0.2
OS	30.0	0.0	69.7	0.0	0.0	0.0	0.3
PS	0.0	0.2	92.1	1.1	4.8	0.0	1.9
RB	42.3	2.0	4.4	8.6	21.3	19.0	2.4
RP	47.1	0.0	52.9	0.0	0.0	0.0	0.0
RT	56.7	3.2	7.0	5.2	17.9	8.5	1.4
SB	54.4	3.3	6.6	5.3	20.5	7.7	2.2
SD	72.1	1.4	2.5	2.5	12.2	5.4	3.9
SL	73.9	2.5	5.4	1.3	2.6	0.0	14.2
SP	62.4	2.5	4.7	1.3	15.1	12.4	1.7
ST	26.5	9.6	52.7	2.5	5.8	0.0	2.9
TE	3.4	78.9	0.0	4.7	13.0	0.0	0.0
TN	0.0	89.5	1.8	8.1	0.0	0.0	0.7
TS	49.6	2.1	8.4	3.6	8.5	24.3	3.6
UH	57.4	3.6	8.4	2.8	16.3	9.5	1.9
VF	0.0	99.8	0.0	0.0	0.0	0.0	0.2
VK	19.7	0.0	67.9	0.0	12.2	0.0	0.3
WH	60.5	2.7	7.1	3.9	18.1	5.0	2.8
WM	0.0	0.0	0.0	0.0	0.0	0.0	100.0
WS	25.1	59.8	7.9	1.2	5.9	0.0	0.1
WW	21.3	10.0	61.8	0.4	0.4	0.0	6.1

**Table 6:** Discharge points of combined sewer catchments and the share of sewage discharged to different surface water sections

Short name	Name	Discharge point	Receiving water	Share
BLN1	Berlin I	BLN1a	Kupfergraben	6.4
		BLN1b	Landwehrkanal	91.7
		BLN1c	Spree	1.9
BLN2	Berlin II	BLN2a	Kupfergraben	2.6
		BLN2b	Landwehrkanal	96.2
		BLN2c	Spree	1.2
BLN3	Berlin III	BLN3a	Kupfergraben	15.9
		BLN3b	Landwehrkanal	49.7
		BLN3c	Spree	34.4
BLN4	Berlin IV	BLN4a	Berlin-Spandauer-Schifffahrtskanal	64.0
		BLN4b	Panke	3.5
		BLN4c	Spree	32.5
BLN5	Berlin V	BLN5	Spree	100
BLN7	Berlin VII	BLN7	Landwehrkanal	100
BLN8	Berlin VIII	BLN8a	Berlin-Spandauer-Schifffahrtskanal	1.0
		BLN8b	Spree	99.0
BLN9	Berlin IX	BLN9	Berlin-Spandauer-Schifffahrtskanal	100
BLN10	Berlin X	BLN10a	Panke	98.0
		BLN10b	Spree	2.0
BLN11	Berlin XI	BLN11	Spree	100
BLN12	Berlin XII	BLN12	Spree	100
CHB1	Charlottenburg I	CHB1	Landwehrkanal	26.7
NKN1	Neuköln I	NKN1	Neuköllner Schifffahrtskanal	89.5
NKN2	Neuköln II	NKN2	Neuköllner Schifffahrtskanal	100
WIL	Wilmersdorf	WIL	Landwehrkanal	100

**Table 7:** Discharge points of separate sewer catchments and the share of sewage discharged to different surface water sections

Short name	Name	Discharge point	Receiving water	Share
AES	Alte Erpe, Alte Spree	AES	Alte Erpe	100
BKE	Bäke	BKE	Bäke	100
BVK	Britzer Verbindungskanal	BVK	Britzer Verbindungskanal	100
DHM	Dahme	DHM	Dahme	100
FAS	Fauler See	FAS	Fauler See	100
KLS	Krumme Lanke, süd	KLS	Krumme Lanke	100
KNS	Kanäle nördlich der Spree	KNS	Kanäle nördlich der Spree	100
KSS	Kanäle südlich der Spree	KSS	Neuköllner Schifffahrtskanal and Landwehrkanal	100
LKF	Landwehrkanal Flutgraben	LKF	Landwehrkanal and Flutgraben	100
LSK	Langer See Köpenick	SKP	Langer See	100
MSP	Müggelspree, upstream Köpenick	MSP	Müggelspree	100
NPK	nördliche Panke	NPK	nördliche Panke	100
NSK	Neuköllner Schifffahrtskanal	NSK	Neuköllner Schifffahrtskanal	100
OBS	Obersee	OBS	Obersee	100
OHV	Oberhavel (Tegeler See bis Schleuse Spandau)	OHV	Oberhavel	100
RBB	Rummelburger Bucht	RBB	Rummelburger Bucht	100
SCS	Schlachtensee	SCS	Schlachtensee	100
STF	Stadtspree, Flutgraben	STF	Stadtspree	100
SIJ	Stadtspreee, Insel der Jugend	SIJ	Stadtspree	100
SPN	Spandau	SPN	westlicher Abzugsgraben	100
STK	Stichkanal	STK	Stichkanal	100
STM	Stadtspree bis Mündung	STM	Stadtspree	100
STS	Stoessensee	STS	Stoessensee	100
TGH	Tegelersee, Borsighafen	TGH	Tegelersee/Borsighafen	100
UBH	Urbanhafen	UBH	Urbanhafen	100
WUH	Wuhle	WUH	Wuhle	100

**Table 8:** HPLC gradient at 0.4 ml/min flow rate

Time (min)	Eluent A (%)	Eluent B (%)
0	95	5
3.0	95	5
4.5	5	95
6.0	5	95

A post-time of 4.5 min was included

**Table 9:** Mass spectrum operating settings

Gas temperature (°C)	170
Gas flow (l min^−1^)	11
Nebulizer (psi)	55
Sheath gas heater (°C)	300
Sheath gas flow (l min^−1^)	11
Capillary (V)	2000
Nozzle (V)	500
Delta EMV ( +)	400

**Table 10:** Substance-specific parameters of the LC–MS/MS method for nicotine and nicotine-D4

Compound name	Precursor ion	Product ion	Fragmentor	Collision energy
Nicotine	163.1	132.1	109	14
Nicotine	163.1	130.1	109	22
Nicotine	163.1	117.1	109	30
Nicotine	163.1	84	109	20
Nicotine-D4	167.2	134.1	109	22
Nicotine-D4	167.2	121.1	109	30
Nicotine-D4	167.2	84	109	24
